# Supplementary material for: Tolerance for democratic norm violations increases when sincerity replaces accuracy as a marker of honesty
Source: Commun Psychol. 2026 Feb 4;4:45. doi: 10.1038/s44271-026-00407-w (PMC12979203; doi:10.1038/s44271-026-00407-w)
Supplement: Supplementary file 2 — Online supplement [file 44271_2026_407_MOESM2_ESM.pdf]

Online supplement for 'Tolerance for democratic norm violations increases when sincerity replaces accuracy as a marker of honesty"

Kiia Jasmin Alexandra Huttunen [1] and Stephan Lewandowsky[1,2]

1. University of Bristol

Bristol

UK

2. University of Potsdam

Potsdam

Germany

Author Note

Kiia Huttunen https://orcid.org/0000-0003-3515-5064; Stephan Lewandowsky https://orcid.org/0000-0003-1655-2013

Online supplement for 'Tolerance for democratic norm violations increases when sincerity replaces accuracy as a marker of honesty"

Contents

Supplementary notes 1: Experiment 1: Multiple regression **3**

Supplementary notes 2: Confirmatory factor analyses **3**

Supplementary notes 3: Welch's f-test results for Experiments 2 and 3 **4**

Supplementary notes 4: Measuring tolerance of norm violations **5**

Supplementary notes 5: Full distribution of participants per experimental condition for all experiments **7**

Supplementary notes 6: Intention to Treat ANOVA tables **10**

Supplementary notes 7: The Epistemic Evidence Intuition Scale [E2IS; 10] **22**

Supplementary notes 1: Experiment 1: Multiple regression

In Experiment 1 we preregistered an exploratory analysis to examine whether the traits of conspiracist mentality and proclivity for non-normative political engagement could explain a participants' preference for sincerity or accuracy. Two multiple regressions were run to predict preference for accuracy or sincerity from conspiracist mentality (as measured with the Conspiracist Mentality Questionnaire by Bruder et al. [1]) and non-normative political engagement (as measured with the questionnaire developed by Imhoff, Dieterle, and Lamberty [2]). The first multiple regression for accuracy revealed that the overall model was not significant, $F(2, 235) = 2.863$, $p = 0.06$, $R^2 = 0.024$. Of the individual predictors, while conspiracist mentality was not a significant component, $t = 1.17$, $p = 0.24$, non-normative political behaviour was, $t = -2.2$, $p = 0.028$. This indicated that the more supportive of non-normative political behaviour a person was, the less likely they were to prefer accuracy. The second multiple regression for sincerity showed that the overall model was significant, $F(2, 235) = 8.165$, $p < 0.001$, $R^2 = 0.065$. Conspiracist mentality was a significant predictor ($t = 4.04$, < 0.001) whereas non-normative political engagement was not, $t = -0.36$, $p = 0.72$. Thus, when people scored higher on conspiracist mentality they were more likely to prefer sincerity.

Supplementary notes 2: Confirmatory factor analyses

In Experiments 2, 3, and 4, we conducted a Confirmatory Factor Analysis (CFA) to test for a two factor structure, as established in the EFA in Experiment 1. The data was analysed using the "lavaan" package in R [3, 4] with maximum likelihood estimation to fit the model.

The CFA results from Experiment 2 indicated mixed results for the fit of the model, $\chi^2(26) = 183.497, p < .001, CFI = 0.933, TLI = 0.908, RMSEA = 0.144, 90\%CI = [.124, .163], SRMR = .048$. While the CFI and TLI values are acceptable, the RMSEA value is high and indicates a poor fit. We accepted the model because its fit had been established in Experiment 1. For the "perceived honesty" factor the factor loadings were all

significant and as follows: accuracy (.736), authenticity (.899), competence (.639), genuineness (.822), sincerity (.872), honesty (.939) and truthfulness (.923). For "likeability" factor, the loadings were .730 for warmth and .847 for likeability.

For Experiment 3, the CFA results again indicated a mixed fit, $\chi^2(26) = 222.573, p < .001, CFI = 0.944, TLI = 0.923, RMSEA = 0.130, 90\%CI = [.114, .146], SRMR = .049$. While the high RMSEA value indicated a poor fit, as the CFI and TLI values were acceptable we therefore again accepted the model. For the "perceived honesty" factor the factor loadings were all significant and as follows: accuracy (.792), authenticity (.845), competence (.596), genuineness (.859), sincerity (.890), honesty (.909) and truthfulness (.922). For "likeability" factor, the loadings were .826 for warmth and .851 for likeability.

The CFA results for Experiment 4 also indicated a mixed fit, $\chi^2(26) = 188.720, p < .001, CFI = 0.963, TLI = 0.949, RMSEA = 0.106, 90\%CI = [.092, .120], SRMR = .036$. Although the high RMSEA value indicated a poor fit, the CFI and TLI values were acceptable. We again accepted the model. For the "perceived honesty" factor the factor loadings were all significant and as follows: accuracy (.816), authenticity (.889), competence (.623), genuineness (.816), sincerity (.876), honesty (.926), and likeability (.920). For "likeability" factor, the loadings were .785 for warmth and .833 for likeability.

Supplementary notes 3: Welch's f-test results for Experiments 2 and 3

Levene's test for homogeneity of variance was discovered to be violated in Experiments 2 and 3 for incitement and dishonesty, and in Experiment 4 for dishonesty and likeability. Further, dishonesty had a positive skew and was mildly leptokurtic in all four experiments. While parametric ANOVAs are generally robust enough for such minor deviations ([e.g., 5, 6, 7, 8]), some such as Delacre et al. [9] argue that Welch's f -test should be utilised instead. Thus, to ensure the robustness of our pre-registered and hypothesised findings, we report the results of these for the variables where there were violations of homogeneity and/or normality.

Looking first at Experiment 2 and incitement, we find that the main effect of perspective on incitement, Welch's $W(1, 185.9) = 12.332$, $p < 0.001$ and dishonesty Welch's $W(1, 144.9) = 12.067$, $p < 0.001$ are significant. In addition, the main effect of truthfulness on incitement Welch's $W(1, 185.9) = 16.437$, $p < 0.001$ is significant.

Similarly for Experiment 3, the main effects of perspective on incitement Welch's $W(1, 369.1) = 21.749$, $p < 0.001$, and on dishonesty Welch's $W(1, 369.1) = 7.428$, $p < 0.001$ were significant as was the main effect of truthfulness on incitement, Welch's $W(1, 369.1) = 7.428$, $p = 0.007$.

Lastly, in Experiment 4 the main effect of perspective on dishonesty, Welch's $W(1, 448.2) = 18.564$, $p < 0.001$, was significant. In addition, for likeability the main effects normativity Welch's $W(1, 488.1) = 1.751$, $p < 0.001$ and truthfulness Welch's $W(1, 369.1) = 10.230$, $p = 0.002$, as well as the interaction between perspective and truthfulness, Welch's $W(1, 488.1) = 7.290$, $p = 0.007$, and a three-way interaction perspective × truthfulness × normativity Welch's $W(1, 488.1) = 4.823$, $p = 0.03$ were all significant.

These results show that as hypothesised, despite the minor violations of homogeneity and normativity, the ANOVA results presented in the main manuscript are robust.

Supplementary notes 4: Measuring tolerance of norm violations

The following nine items were used to measure participants' tolerance for norm-violations in all of the four Experiments. Approval was rated on a Likert scale from strongly disagree (1) to strongly agree (7). Participants were instructed as follows:

"Bearing in mind your perspective of Sam/Taylor, please answer the following questions."

Incitement

1. "The demonstrations calling for the referendum were necessary to display public support."

2. "The public should support the Prime Minister in implementing policies - with all means necessary."

3. "The public should avoid getting utilised by the Prime Minister for his political goals."

Dishonesty

4. "Politics demands a certain degree of deception. I therefore approve of the Prime Minister's actions to achieve his goals."

5. "If necessary, politicians should lie to implement policies that are good for the country."

6. "Politicians should serve as a role model for citizens' behaviour. Dishonesty is not compatible with this role."

Expansion of power

7. "The parliament should have more discretion on which controversial matters to decide amongst themselves and which ones to put to a referendum."

8. "The Prime Minister does not have enough power to implement his policies. If an opportunity emerges to expand power, he should take it."

9. "If a Prime Minister is working for the well-being of the country, he should not be limited in his actions."

Supplementary notes 5: Full distribution of participants per experimental condition for all experiments

Table S1

Distribution of participants in experimental conditions

Experiment	Condition	n
**Experiment 1**		
	Belief-speaking (BS)	112
	Fact-speaking (FS)	126
	FS, Norm Violation, No Lies	39
	FS, Norm Violation, Lies	34
	FS, Normative Behaviour, No Lies	25
	FS, Normative Behaviour, Lies	28
	BS, Norm Violation, No Lies	38
	BS, Norm violation, Lies	25
	BS, Normative Behaviour, No Lies	26
	BS, Normative Behaviour, Lies	23
**Experiment 2**		
	Belief-speaking (BS)	132
	Fact-speaking (FS)	162
	FS, Norm Violation, No Lies	32
	FS, Norm Violation, Lies	22
	FS, Normative Behaviour, No Lies	33
	FS, Normative Behaviour, Lies	45
	BS, Norm Violation, No Lies	49
	BS, Norm violation, Lies	42
	BS, Normative Behaviour, No Lies	40

Table S1

Distribution of participants in experimental conditions (continued)

Experiment	Condition	n
	BS, Normative Behaviour, Lies	31
**Experiment 3**		
	Belief-speaking (BS)	261
	Fact-speaking (FS)	297
	FS, Norm Violation, No Lies	74
	FS, Norm Violation, Lies	83
	FS, Normative Behaviour, No Lies	63
	FS, Normative Behaviour, Lies	77
	BS, Norm Violation, No Lies	66
	BS, Norm violation, Lies	60
	BS, Normative Behaviour, No Lies	60
	BS, Normative Behaviour, Lies	75
**Experiment 4**		
	Belief-speaking (BS)	261
	Fact-speaking (FS)	297
	FS, Norm Violation, No Lies	74
	FS, Norm Violation, Lies	83
	FS, Normative Behaviour, No Lies	63
	FS, Normative Behaviour, Lies	77
	BS, Norm Violation, No Lies	66
	BS, Norm violation, Lies	60
	BS, Normative Behaviour, No Lies	60
	BS, Normative Behaviour, Lies	75

Table S1

Distribution of participants in experimental conditions (continued)

Experiment	Condition	n

Note. E1 n = 238; E2 n = 294; E3 n = 449; E4 n = 558.

Supplementary notes 6: Intention to Treat ANOVA tables

Table S2

Experiment 1: Intention to Treat ANOVA results

									95% CI	
DV	Effect	df	SS	MS	F	p	BH α	η^2_p	Lower	Upper
**Incitement**										
	Truthfulness	1	18.21	18.21	15.73	< **0.001**	**0.013**	0.05	0.02	1.00
	Normativity		3.61	3.61	3.12	0.08	0.044	0.01	0.00	1.00
	Perspective		14.64	14.64	12.65	< **0.001**	**0.019**	0.01	0.01	1.00
	Truthfulness × Normativity		0.99	0.99	0.85	0.36	0.050	0.04	0.00	1.00
	Truthfulness × Perspective		1.11	1.11	0.96	0.33	0.025	< 0.01	0.00	1.00
	Normativity × Perspective		2.66	2.66	2.30	0.13	0.031	< 0.01	0.00	1.00
	Truthfulness × Normativity × Perspective		0.15	0.15	0.13	0.72	0.038	< 0.01	0.00	1.00
	Residuals	286	331.04	1.16						
**Dishonesty**										
	Truthfulness	1	5.32	5.32	3.96	0.05	0.025	0.01	0.00	1.00
	Normativity		0.98	0.98	0.73	0.39	0.019	< 0.01	0.00	1.00
	Perspective		18.82	18.82	14.03	< **0.001**	**0.013**	0.05	0.01	1.00
	Truthfulness × Normativity		0.13	0.13	0.10	0.76	0.044	< 0.01	0.00	1.00
	Truthfulness × Perspective		4.69	4.69	3.50	0.06	0.050	0.01	0.00	1.00
	Normativity × Perspective		0.28	0.28	0.21	0.65	0.031	< 0.01	0.00	1.00
	Truthfulness × Normativity × Perspective		1.47	1.47	1.10	0.30	0.038	< 0.01	0.00	1.00

Continued on next page

Table S2

Experiment 1: Intention to Treat ANOVA results (continued)

DV	Effect	df	SS	MS	F	p	BH α	η^2_p	95% CI Lower	95% CI Upper
	Residuals	286	383.55	1.34						
**Expansion of power**										
	Truthfulness	1	6.41	6.41	4.09	**0.04**	0.025	0.01	0.00	1.00
	Normativity		0.05	0.03	0.86	0.88	0.031	< 0.01	0.00	1.00
	Perspective		25.11	16.02	< **0.001**	< **0.001**	0.013	0.05	0.02	1.00
	Truthfulness × Normativity		0.04	0.02	0.88	0.88	0.050	< 0.01	0.00	1.00
	Truthfulness × Perspective		5.72	3.65	0.06	0.10	0.038	0.01	0.00	1.00
	Normativity × Perspective		7.44	4.75	0.03	0.10	0.019	0.02	0.00	1.00
	Truthfulness × Normativity × Perspective		0.24	0.15	0.70	0.88	0.044	< 0.01	0.00	1.00
	Residuals	286	448.17	1.57						
**Perceived honesty**										
	Truthfulness	1	46.08	46.08	22.49	< **0.001**	**0.013**	0.07	0.03	1.00
	Normativity		18.95	18.95	9.25	< **0.001**	**0.019**	0.03	0.01	1.00
	Perspective		0.03	0.03	0.01	0.91	0.038	< 0.01	0.00	1.00
	Truthfulness × Normativity		1.28	1.28	0.62	0.43	0.50	< 0.01	0.00	1.00
	Truthfulness × Perspective		0.11	0.11	0.05	0.82	0.031	< 0.01	0.00	1.00
	Normativity × Perspective		0.31	0.31	0.15	0.70	0.044	< 0.01	0.00	1.00
	Truthfulness × Normativity × Perspective		1.40	1.40	0.68	0.41	0.025	< 0.01	0.00	1.00

Continued on next page

Table S2

Experiment 1: Intention to Treat ANOVA results (continued)

									95% CI	
DV	Effect	df	SS	MS	F	p	BH α	η^2_p	Lower	Upper
	Residuals	286	585.97	2.05						
**Likeability**										
	Truthfulness	1	17.01	17.01	9.73	< **0.001**	**0.019**	0.03	0.00	1.00
	Normativity		48.69	48.69	27.84	< **0.001**	**0.013**	0.09	0.04	1.00
	Perspective		0.18	0.18	0.10	0.75	0.0.44	< 0.01	0.00	1.00
	Truthfulness × Normativity		0.83	0.83	0.47	0.49	0.050	< 0.01	0.00	1.00
	Truthfulness × Perspective		0.01	0.01	0.01	0.94	0.038	< 0.01	0.00	1.00
	Normativity × Perspective		1.41	1.41	0.81	0.37	0.025	< 0.01	0.00	1.00
	Truthfulness × Normativity × Perspective	1	0.61	0.61	0.35	0.55	0.031	< 0.01	0.00	1.00
	Residuals	286	580.59	1.73						

Note. BH stands for Benjamini–Hochberg. This table presents unfiltered ANOVA results from Experiment 1.

Listwise N = 294.

Table S3

Experiment 2: Intention to Treat ANOVA results

									95% CI	
DV	Effect	df	SS	MS	F	p	BH α	η^2_{p}	Lower	Upper
**Incitement**										
	Truthfulness	1	15.81	15.81	13.30	< **0.001**	**0.013**	0.04	0.01	1.00
	Normativity		5.27	5.27	4.44	**0.04**	0.038	0.01	0.00	1.00
	Perspective		6.36	6.36	5.35	**0.02**	0.031	0.02	0.00	1.00
	Truthfulness × Normativity		2.30	2.30	1.93	0.17	0.019	< 0.01	0.00	1.00
	Truthfulness × Perspective		0.23	0.23	0.19	0.66	0.044	< 0.01	0.00	1.00
	Normativity × Perspective		1.32	1.32	1.11	0.29	0.025	< 0.01	0.00	1.00
	Truthfulness × Normativity × Perspective		0.00	0.00	0.00	0.98	0.050	< 0.01	0.00	1.00
	Residuals	336	399.30	1.19						
**Dishonesty**										
	Truthfulness	1	3.09	3.09	2.31	0.13	0.013	< 0.01	0.00	1.00
	Normativity		0.58	0.58	0.43	0.51	0.031	< 0.01	0.00	1.00
	Perspective		14.81	14.81	11.10	< **0.001**	**0.050**	0.03	0.01	1.00
	Truthfulness × Normativity		1.77	1.77	1.33	0.25	0.025	< 0.01	0.00	1.00
	Truthfulness × Perspective		1.90	1.90	1.42	0.23	0.019	< 0.01	0.00	1.00
	Normativity × Perspective		0.00	0.00	0.00	0.98	0.044	< 0.01	0.00	1.00
	Truthfulness × Normativity × Perspective		4.75	4.75	3.56	0.06	0.038	0.01	0.00	1.00
	Residuals	336	448.46	1.33						

Continued on next page

Table S3

Experiment 2: Intention to Treat ANOVA results (continued)

									95% CI	
DV	Effect	df	SS	MS	F	p	BH α	η^2_p	Lower	Upper
**Expansion of power**										
	Truthfulness	1	3.68	3.68	2.32	0.13	0.013	< 0.01	0.00	1.00
	Normativity		0.12	0.12	0.08	0.78	0.025	< 0.01	0.00	1.00
	Perspective		10.80	10.80	6.81	**0.01**	**0.050**	0.02	0.00	1.00
	Truthfulness × Normativity		0.30	0.30	0.19	0.66	0.044	< 0.01	0.00	1.00
	Truthfulness × Perspective		2.37	2.37	1.50	0.22	0.031	< 0.01	0.00	1.00
	Normativity × Perspective		5.83	5.83	3.68	0.06	0.019	0.01	0.00	1.00
	Truthfulness × Normativity × Perspective		0.53	0.53	0.33	0.56	0.038	< 0.01	0.00	1.00
	Residuals	336	532.75	1.59						
**Perceived honesty**										
	Truthfulness	1	34.22	34.22	16.97	< **0.001**	**0.013**	0.05	0.02	1.00
	Normativity		13.68	13.68	6.78	**0.01**	**0.019**	0.02	0.00	1.00
	Perspective		6.45	6.45	3.20	0.07	0.038	< 0.001	0.00	1.00
	Truthfulness × Normativity		1.66	1.66	0.82	0.36	0.050	< 0.01	0.00	1.00
	Truthfulness × Perspective		0.27	0.27	0.13	0.71	0.025	< 0.01	0.00	1.00
	Normativity × Perspective		0.46	0.46	0.23	0.63	0.044	< 0.01	0.00	1.00
	Truthfulness × Normativity × Perspective		1.38	1.38	0.69	0.41	0.044	< 0.01	0.00	1.00
	Residuals	336	677.34	2.02						

Continued on next page

Table S3

Experiment 2: Intention to Treat ANOVA results (continued)

									95% CI	
DV	Effect	df	SS	MS	F	p	BH α	η^2_p	Lower	Upper
**Likeability**										
	Truthfulness	1	14.03	14.03	8.12	< **0.001**	**0.019**	0.02	0.00	1.00
	Normativity		50.86	50.86	29.44	< **0.001**	**0.013**	0.08	0.04	1.00
	Perspective		0.69	0.69	0.40	0.53	0.044	< 0.01	0.00	1.00
	Truthfulness × Normativity		3.91	3.91	2.26	0.13	0.031	< 0.01	0.00	1.00
	Truthfulness × Perspective		0.27	0.27	0.15	0.70	0.038	< 0.01	0.00	1.00
	Normativity × Perspective		0.64	0.64	0.37	0.54	0.025	< 0.01	0.00	1.00
	Truthfulness × Normativity × Perspective		0.41	0.41	0.24	0.63	0.050	< 0.01	0.00	1.00
	Residuals	336	580.59	1.73						

Note. BH stands for Benjamini-Hochberg. This table presents unfiltered ANOVA results from Experiment 2.

Listwise N = 346.

Table S4

Experiment 3: Intention to Treat ANOVA results

									95% CI	
DV	Effect	df	SS	MS	F	p	BH α	η^2_p	Lower	Upper
**Incitement**										
	Truthfulness	1	12.30	12.30	10.22	< **0.001**	**0.031**	0.02	0.00	1.00
	Normativity		0.11	0.11	0.09	0.76	0.025	< 0.01	0.00	1.00
	Perspective		16.29	16.29	13.53	< **0.001**	**0.013**	0.03	0.01	1.00
	Truthfulness × Normativity		4.86	4.86	4.04	**0.05**	0.019	< 0.01	0.00	1.00
	Truthfulness × Perspective		0.20	0.20	0.17	0.68	0.038	< 0.01	0.00	1.00
	Normativity × Perspective		0.06	0.06	0.05	0.82	0.044	< 0.01	0.00	1.00
	Truthfulness × Normativity × Perspective		0.00	0.00	0.00	0.96	0.050	< 0.01	0.00	1.00
	Residuals	475	572.07	1.20						
**Dishonesty**										
	Truthfulness	1	1.21	1.21	0.83	0.36	0.031	< 0.01	0.00	1.00
	Normativity		5.32	5.32	3.64	0.06	0.050	< 0.01	0.00	1.00
	Perspective		11.68	11.68	7.99	< **0.001**	**0.013**	0.02	0.00	1.00
	Truthfulness × Normativity		0.24	0.24	0.16	0.69	0.025	< 0.01	0.00	1.00
	Truthfulness × Perspective		2.69	2.69	1.84	0.18	0.019	< 0.01	0.00	1.00
	Normativity × Perspective		0.01	0.01	0.01	0.94	0.044	< 0.01	0.00	1.00
	Truthfulness × Normativity × Perspective		0.60	0.60	0.41	0.52	0.038	< 0.01	0.00	1.00
	Residuals	475	694.36	1.46						

Continued on next page

Table S4

Experiment 3: Intention to Treat ANOVA results (continued)

									95% CI	
DV	Effect	df	SS	MS	F	p	BH α	η^2_{p}	Lower	Upper
**Expansion of power**										
	Truthfulness	1	12.81	12.81	8.17	< **0.001**	**0.019**	0.02	0.00	1.00
	Normativity		3.45	3.45	2.20	0.14	0.044	< 0.01	0.00	1.00
	Perspective		19.96	19.96	12.73	< **0.001**	**0.013**	0.03	0.01	1.00
	Truthfulness × Normativity		0.93	0.93	0.59	0.44	0.025	< 0.01	0.00	1.00
	Truthfulness × Perspective		0.70	0.70	0.44	0.51	0.038	< 0.01	0.00	1.00
	Normativity × Perspective		0.97	0.97	0.62	0.43	0.031	< 0.01	0.00	1.00
	Truthfulness × Normativity × Perspective		0.02	0.02	0.01	0.92	0.050	< 0.01	0.00	1.00
	Residuals	475	745.24	1.57						
**Perceived honesty**										
	Truthfulness	1	66.50	66.50	33.02	< **0.001**	**0.019**	0.06	0.03	1.00
	Normativity		79.19	79.19	39.32	< **0.001**	**0.025**	0.08	0.04	1.00
	Perspective		18.56	18.56	9.21	< **0.001**	**0.013**	0.02	0.00	1.00
	Truthfulness × Normativity		0.26	0.26	0.13	0.72	0.044	< 0.01	0.00	1.00
	Truthfulness × Perspective		5.53	5.53	2.74	0.10	0.050	< 0.01	0.00	1.00
	Normativity × Perspective		11.61	11.61	5.77	**0.02**	**0.03**	0.01	0.00	1.00
	Truthfulness × Normativity × Perspective		1.74	1.74	0.87	0.35	0.038	< 0.01	0.00	1.00
	Residuals	475	956.73	2.01						

Continued on next page

Table S4

Experiment 3: Intention to Treat ANOVA results (continued)

									95% CI	
DV	Effect	df	SS	MS	F	p	BH α	η^2_p	Lower	Upper
**Likeability**										
	Truthfulness	1	8.45	8.45	3.87	0.05	0.031	< 0.01	0.00	1.00
	Normativity		58.15	58.15	26.64	< **0.001**	**0.013**	0.05	0.03	1.00
	Perspective		0.32	0.32	0.15	0.70	0.019	< 0.01	0.00	1.00
	Truthfulness × Normativity		0.97	0.97	0.44	0.51	0.044	< 0.01	0.00	1.00
	Truthfulness × Perspective		3.69	3.69	1.69	0.19	0.038	< 0.01	0.00	1.00
	Normativity × Perspective		10.19	10.19	4.67	0.03	0.025	< 0.01	0.00	1.00
	Truthfulness × Normativity × Perspective		0.19	0.19	0.09	0.77	0.050	< 0.01	0.00	1.00
	Residuals	475	1037.04	2.18						

Note. BH stands for Benjamini-Hochberg. This table presents unfiltered ANOVA results from Experiment 3.

Listwise $N = 483$.

Table S5

Experiment 4: Intention to Treat ANOVA results

									95% CI	
DV	Effect	df	SS	MS	F	p	BH α	η^2_p	Lower	Upper
**Incitement**										
	Truthfulness	1	45.59	45.59	36.660	< **0.001**	**0.007**	0.06	0.03	1.00
	Normativity		6.08	6.08	4.89	**0.027**	**0.021**	< 0.01	0.00	1.00
	Perspective		26.27	26.27	21.13	< **0.001**	**0.014**	0.03	0.01	1.00
	Truthfulness × Normativity		0.470	0.470	0.378	0.539	0.050	< 0.01	0.00	1.00
	Truthfulness × Perspective		0.701	0.701	0.564	0.453	0.036	< 0.01	0.00	1.00
	Normativity × Perspective		0.010	0.010	0.008	0.927	0.044	< 0.01	0.00	1.00
	Truthfulness × Normativity × Perspective		0.555	0.555	0.446	0.504	0.038	<0.01	0.00	1.00
	Residuals	592	736.21	1.24						
**Dishonesty**										
	Truthfulness	1	0.21	0.21	0.13	0.72	0.05	< 0.01	0.00	1.00
	Normativity		0.21	0.21	0.14	0.71	0.04	< 0.01	0.00	1.00
	Perspective		28.87	28.87	18.25	< **0.001**	**0.01**	0.03	0.00	1.00
	Truthfulness × Normativity		1.03	1.03	0.65	0.42	0.03	< 0.01	0.00	1.00
	Truthfulness × Perspective		1.88	1.88	1.19	0.28	0.01	< 0.01	0.00	1.00
	Normativity × Perspective		0.62	0.62	0.39	0.53	0.04	< 0.01	0.00	1.00
	Truthfulness × Normativity × Perspective		1.20	1.20	0.76	0.38	0.02	< 0.01	0.00	1.00
	Residuals	592	936.31	1.58						

Continued on next page

Table S5

Experiment 4: Intention to Treat ANOVA results (continued)

DV	Effect	df	SS	MS	F	p	BH α	η^2_p	95% CI Lower	95% CI Upper
**Expansion of Power**										
	Truthfulness	1	17.88	17.88	13.04	< **0.001**	**0.01**	0.02	0.01	1.00
	Normativity		0.00	0.00	0.001	0.98	0.04	< 0.01	0.00	1.00
	Perspective		28.46	28.46	20.76	< **0.001**	**0.01**	0.03	0.01	1.00
	Truthfulness × Normativity		8.06	8.06	5.88	0.02	0.02	< 0.01	0.00	1.00
	Truthfulness × Perspective		1.65	1.65	1.20	0.27	0.03	< 0.01	0.00	1.00
	Normativity × Perspective		0.00	0.00	0.00	0.99	0.05	< 0.01	0.00	1.00
	Truthfulness × Normativity × Perspective		0.17	0.17	0.12	0.73	0.04	< 0.01	0.00	1.00
	Residuals	592	811.53	1.37						
**Perceived Honesty**										
	Truthfulness	1	123.79	123.79	66.05	< **0.001**	**0.01**	0.10	0.07	1.00
	Normativity		39.63	39.63	21.14	< **0.001**	**0.01**	0.03	0.01	1.00
	Perspective		29.35	29.35	15.66	< **0.001**	**0.02**	0.03	0.01	1.00
	Truthfulness × Normativity		1.27	1.27	0.68	0.41	0.05	< 0.01	0.00	1.00
	Truthfulness × Perspective		4.298	4.298	2.293	0.130	0.044	< 0.01	0.00	1.00
	Normativity × Perspective		11.84	11.84	6.32	**0.01**	**0.04**	0.01	0.00	1.00
	Truthfulness × Normativity × Perspective		18.76	18.76	10.01	**0.002**	**0.031**	0.02	0.00	1.00
	Residuals	592	1109.60	1.87						

Continued on next page

Table S5

Experiment 4: Intention to Treat ANOVA results (continued)

									95% CI	
DV	Effect	df	SS	MS	F	p	BH α	η^2_p	Lower	Upper
**Likeability**										
	Truthfulness	1	19.64	19.64	10.45	< **0.001**	**0.01**	0.02	0.00	1.00
	Normativity		36.70	36.70	19.52	< **0.001**	**0.01**	0.03	0.01	1.00
	Perspective		9.85	9.85	5.24	**0.02**	**0.02**	< 0.01	0.00	1.00
	Truthfulness × Normativity		0.01	0.01	0.00	0.96	0.05	< 0.00	0.00	1.00
	Truthfulness × Perspective		8.11	8.11	4.31	**0.04**	**0.03**	< 0.00	0.00	1.00
	Normativity × Perspective		1.19	1.19	0.63	0.43	0.04	< 0.00	0.00	1.00
	Truthfulness × Normativity × Perspective		7.33	7.33	3.90	0.05	0.04	<	0.00	1.00
	Residuals	592	1112.83	1.88						

Note. BH stands for Benjamini-Hochberg. This table presents unfiltered ANOVA results from Experiment 4.

Listwise N = 600.

Supplementary notes 7: The Epistemic Evidence Intuition Scale [E2IS; 10]

We briefly summarize construction of the scale. Data collection was conducted in multiple waves between March and November 2023, beginning in the United Kingdom, followed by Germany, and subsequently including Hungary, Italy, and Spain in parallel. The survey was administered via Positly.com, which was selected as the provider because it enabled recruitment from all five target countries. Compensation followed Positly's standard scheme, comprising a fixed payment to participants and a variable fee determined by the availability of individuals within Positly's participant pool. An overview of demographic characteristics and data collection periods is presented in Table S6.

Table S6

Demographic Statistics of Study 2

	N	M_{age}	SD	Data collection
Germany	888	37.91	13.56	12.07. to 16.08.23
Hungary	1085	34.45	10.91	15.09. to 19.11.23
Italy	1048	39.09	12.52	15.09. to 17.09.23
Spain	1052	37.43	11.32	15.09. to 19.09.23
United Kingdom	1048	39.47	12.65	21.03. to 04.04.23

Note. Because of difficulties in reaching the target sample size in Hungary, the data collection period had to be extended several times. In Germany, the smaller sample size resulted from the limited number of eligible participants in the pool.

A multi-group SEM analysis was conducted to assess the measurement equivalence of the E2IS. Establishing equivalence is a prerequisite for comparing latent means across groups. The procedure follows an iterative sequence, testing successive levels of measurement invariance—configural, metric, scalar, and strict—until model fit deteriorates beyond acceptable thresholds [11]. Configural invariance reflects structural equivalence, meaning

that items measure the same constructs across all groups. Metric invariance adds the condition that factor loadings between observed indicators and latent constructs are identical; without it, estimates of factor variances, covariances, and means may be biased [11]. Scalar invariance is achieved when both factor loadings and intercepts are consistent across groups, which is essential for comparing observed and latent means. Strict invariance, the most stringent level, requires residual variances (i.e., unexplained variances) to be equal across groups [12]. The iterative testing stops once model fit declines beyond recommended thresholds, specifically when the constrained model shows a drop in CFI greater than .01 [13, 14], or increases in RMSEA or SRMR beyond 0.015 and 0.030, respectively [14]. As in Study 1, overall model fit was judged by inspecting CFI (> 0.90), SRMR (< 0.08), and RMSEA (lower bound < 0.05, upper bound < 0.08). Table S7 presents the SEM fit indices for configural, metric, and scalar models.

Table S7

Measurement Equivalence of the E2IS Across Five Countries

Levels of measurement invariance	χ^2	df	SRMR	ΔSRMR	CFI	ΔCFI	RMSEA	ΔRMSEA	90% CI
Configural	3554.73	825	0.058	–	0.919	–	0.057	–	0.055-0.059
Metric	3753.75	897	0.061	0.003	0.915	-0.004	0.056	0.001	0.054-0.058
Scalar	4621.73	957	0.065	0.004	0.891	-0.024	0.065	0.005	0.059-0.063

Note. All models with correlated residuals between two items of the preference for accuracy scale, EP1 and EP4.

The configural model showed acceptable fit on most indices, though the RMSEA (0.057) slightly exceeded the preferred cutoff of 0.05. The metric model, which imposed equality constraints on factor loadings, also fit adequately, with only a marginally elevated RMSEA (0.056). Although the fit declined slightly, the changes remained within acceptable thresholds (Δ CFI < 0.01, Δ RMSEA < 0.015, Δ SRMR < 0.03), supporting metric

invariance across all five countries. In contrast, the scalar model, which additionally constrained intercepts, displayed substantially poorer fit (CFI = 0.891, Δ CFI > 0.01; SRMR = 0.065, Δ SRMR = 0.03; RMSEA = 0.061, Δ RMSEA < 0.015). Accordingly, scalar equivalence could not be established. Table S8 presents the fit of the SEM for both the full sample and the five individual countries.

Table S8

Fit indices for SEM for full sample and individual countries

Construct	χ^2	df	SRMR	CFI	RMSEA	90% CI
Germany	559.42	165	0.059	0.943	0.052	0.047-0.057
Hungary	758.50	165	0.059	0.913	0.058	0.053-0.062
Italy	769.50	165	0.071	0.917	0.059	0.055-0.063
Spain	744.61	165	0.057	0.903	0.058	0.054-0.062
United Kingdom	722.69	165	0.057	0.917	0.057	0.053-0.061

Note. All models with correlated residuals between EP1 and EP4 (Preference for accuracy).

To establish predictive validity, E2IS scores were correlated with various relevant outcome measures. For further information on this, please see Abels and Lewandowsky [10]. An overview of the correlations is shown below in Table S9. The results show that the E2IS is highly correlated with actively open-minded thinking (AOMT), meaning that individuals with an evidence-based understanding of truth are more likely to be open-minded. They are also more likely to engage in normative political actions, whereas those with an intuition-based understanding of truth are more likely to engage in non-normative political actions. These correlations show the instrument's ability to predict politically relevant behavioural intentions.

Table S9

Correlations of E2IS score and Associated Measures Across Countries

	AOMT	PAS	PRS	PEnorm	PEnon
Germany	0.51***	−0.36***	−0.24***	0.12***	−0.34***
Hungary	0.32***	−0.13***	−0.10***	0.04	−0.16***
Italy	0.38***	−0.19***	−0.01	0.15***	−0.38***
Spain	0.29***	−0.06*	−0.11***	0.08**	−0.27***
United Kingdom	0.23***	−0.20***	−0.19***	0.11***	−0.17***

Note. Correlations with E2IS scores (based on factor scores for evidence - intuition). $* \ p < .05$, $** \ p < .01$, $*** \ p < .001$. AOMT = Actively open-minded thinking, PAS = Populist attitudes, PRS = Psychological reactance scale, PEnorm = Normative political engagement, PEnon = Non-normative political engagement.

Supplementary References

[1] Martin Bruder et al. "Measuring Individual Differences in Generic Beliefs in Conspiracy Theories Across Cultures: Conspiracy Mentality Questionnaire". In: *Frontiers in Psychology* 4 (Apr. 2013), p. 225. ISSN: 1664-1078. DOI: 10.3389/FPSYG.2013.00225.

[2] Roland Imhoff, Lea Dieterle, and Pia Lamberty. "Resolving the Puzzle of Conspiracy Worldview and Political Activism: Belief in Secret Plots Decreases Normative but Increases Nonnormative Political Engagement". In: *Social Psychological and Personality Science* 12.1 (2021), pp. 71–79. DOI: 10.1177/1948550619896491. URL: https://journals.sagepub.com/doi/abs/10.1177/1948550619896491.

[3] Yves Rosseel. "lavaan: An R package for structural equation modeling". In: *Journal of Statistical Software* 48 (2012). ISSN: 15487660. DOI: 10.18637/JSS.V048.I02.

[4] R Core Team. *R: A Language and Environment for Statistical Computing*. Vienna, Austria, 2022.

[5] Geoff Norman. "Likert scales, levels of measurement and the "laws" of statistics". In: *Advances in Health Sciences Education* 15.5 (Dec. 2010), pp. 625–632. ISSN: 13824996. DOI: 10.1007/S10459-010-9222-Y/TABLES/1. URL: https://link.springer.com/article/10.1007/s10459-010-9222-y.

[6] Spencer E. Harpe. "How to analyze Likert and other rating scale data". In: *Currents in Pharmacy Teaching and Learning* 7.6 (Nov. 2015), pp. 836–850. ISSN: 1877-1297. DOI: 10.1016/J.CPTL.2015.08.001. URL: https://www.sciencedirect.com/science/article/abs/pii/S1877129715200196.

[7] Suleyman Demir. "Comparison of Normality Tests in Terms of Sample Sizes under Different Skewness and Kurtosis Coefficients". In: *International Journal of Assessment Tools in Education* 2022.2 (2022), pp. 397–409. DOI: 10.21449/ijate.1101295. URL:

https://doi.org/10.21449/ijate.1101295Publishedathttps://ijate.net/https://dergipark.org.tr/en/pub/ijate.

[8] Ulrich Knief and Wolfgang Forstmeier. "Violating the normality assumption may be the lesser of two evils". In: *Behavior Research Methods* 53.6 (Dec. 2021), pp. 2576–2590. ISSN: 15543528. DOI: 10.3758/S13428-021-01587-5. URL: https://link.springer.com/article/10.3758/s13428-021-01587-5.

[9] Marie Delacre et al. "Taking parametric assumptions seriously: Arguments for the use of welch's f-test instead of the classical f-test in one-way ANOVA". In: *International Review of Social Psychology* 32.1 (2020). ISSN: 23978570. DOI: 10.5334/IRSP.198.

[10] Christoph Abels and Stephan Lewandowsky. "Development and Validation of the Evidence Intuition Scale". In: (July 2024). DOI: 10.31234/osf.io/u4xka.

[11] Raymond Luong and Jessica Kay Flake. "Measurement invariance testing using confirmatory factor analysis and alignment optimization: A tutorial for transparent analysis planning and reporting." In: *Psychological Methods* 28.4 (Aug. 2023), pp. 905–924. ISSN: 1939-1463. DOI: 10.1037/met0000441.

[12] William Meredith. "Measurement Invariance, Factor Analysis and Factorial Invariance". In: *Psychometrika* 58.4 (Dec. 1993), pp. 525–543. ISSN: 0033-3123. DOI: 10.1007/BF02294825.

[13] Gordon W. Cheung and Roger B. Rensvold. "Evaluating Goodness-of-Fit Indexes for Testing Measurement Invariance". In: *Structural Equation Modeling: A Multidisciplinary Journal* 9.2 (Apr. 2002), pp. 233–255. ISSN: 1070-5511. DOI: 10.1207/S15328007SEM0902{_}5.

[14] Fang Fang Chen. "Sensitivity of Goodness of Fit Indexes to Lack of Measurement Invariance". In: *Structural Equation Modeling: A Multidisciplinary Journal* 14.3 (July 2007), pp. 464–504. ISSN: 1070-5511. DOI: 10.1080/10705510701301834.
