# Supplementary material for: Tolerance for democratic norm violations increases when sincerity replaces accuracy as a marker of honesty
Source: Commun Psychol. 2026 Feb 4;4:45. doi: 10.1038/s44271-026-00407-w (PMC12979203; doi:10.1038/s44271-026-00407-w)
Supplement: Supplementary file 3 — Online Appendix [file 44271_2026_407_MOESM3_ESM.pdf]

Appendix A

Vignettes

To elicit belief-speaking attitudes and understanding of honesty, participants were presented the following.

"Please take the perspective of Sam when answering the question that will be shown on the next page. This is what Sam has to say about what truth is:

People often disagree about what truth is. Personally, I think that being truthful means always saying what you think, no matter the consequences. There is no other "truth" than being sincere and expressing yourself the way you feel is right. Only when people are sincere, can they really be trusted. And being sincere means genuinely expressing your beliefs, even when you don't have enough evidence to support them."

Alternatively, to elicit fact-speaking understanding of honesty, participants were shown the following.

"Please take the perspective of Taylor when answering the question that will be shown on the next page. This is what Taylor has to say about what truth is:

People often disagree about what truth is. Personally, I think that being truthful means sticking to the facts, regardless of your personal opinions. Opinions, no matter how honestly held, can be far from the truth. Only an argument that is informed by evidence can be trusted. One should be accurate in what one says and refrain from expressing unfounded beliefs."

Appendix B

Descriptive statistics

Table B1
Experiment 1 descriptive statistics

	N	M	SD
Belief-speaking	112		
Fact-speaking	126		
Age	238	45.929	15.796
Authenticity		3.790	1.635
Competence		4.084	1.332
Prestige		4.357	1.257
Genuineness		3.782	1.530
Sincerity		3.714	1.610
Considerateness		3.496	1.422
Warmth		3.723	1.381
Likeability		4.248	1.659
Honesty		3.466	1.732
Truthfulness		3.588	1.691
Accuracy		3.546	1.558
Incitement		4.437	0.763
Dishonesty		3.737	0.833
Expansion		3.930	1.189
Perceived honesty		3.681	1.389
Likeability factor		3.949	1.361

Note. This table presents descriptive statistics from Experiment 1.

Table B2

Experiment 2 descriptive statistics

	N	M	SD
Belief-speaking	132		
Fact-speaking	162		
Age	294	41.895	12.424
Authenticity		4.235	1.672
Competence		4.735	1.369
Genuineness		4.092	1.678
Sincerity		4.122	1.685
Warmth		3.959	1.459
Likeability		4.313	1.610
Honesty		3.952	1.912
Truthfulness		3.946	1.828
Accuracy		4.112	1.433
Expansion		3.712	1.297
Dishonesty		2.155	1.190
Incitement		3.637	1.127
Perceived honesty		4.127	1.494
Likeability factor		4.158	1.393

Note. This table presents descriptive statistics from Experiment 2.

Table B3

Experiment 3 descriptive statistics

	N	M	SD
Belief-speaking	212		

Table B3

Experiment 3 descriptive statistics (continued)

	N	M	SD
Fact-speaking	237		
Age	449	46.227	15.693
Authentic		3.880	1.784
Competence		4.272	1.504
Genuineness		3.800	1.772
Sincerity		3.702	1.800
Warmth		3.581	1.566
Likeability		3.993	1.721
Honesty		3.644	1.907
Truthfulness		3.644	1.915
Accuracy		3.641	1.599
Expansion		3.790	1.278
Dishonesty		2.138	1.248
Incitement		3.625	1.114
Perceived honesty		3.767	1.565
Likeability		3.813	1.530

Note. This table presents descriptive statistics from Experiment 3.

Table B4

Experiment 4 descriptive statistics

	N	M	SD
Belief-speaking	261		
Fact-speaking	297		

Table B4

Experiment 4 descriptive statistics (continued)

	N	M	SD
Age	558	42.584	14.452
Authenticity		3.692	1.732
Competence		4.290	1.414
Genuineness		3.720	1.719
Sincerity		3.599	1.733
Warmth		3.511	1.446
Likeability		3.943	1.670
Honesty		3.518	1.903
Truthfulness		3.525	1.842
Accuracy		3.597	1.511

Note. This table presents descriptive statistics from Experiment 4.

Appendix C

Full per protocol ANOVA tables for all experiments

Table C1

Experiment 1: Full per protocol ANOVA results

									95% CI	
DV	Effect	df	SS	MS	F	p	BH α	η^2_p	Lower	Upper
**Incitement**										
	Truthfulness	1	5.01	5.01	8.92	**0.003**	**0.019**	0.04	0.01	1.00
	Normativity		0.65	0.65	1.16	0.283	0.038	< 0.01	0.00	1.00
	Perspective		1.86	1.86	3.31	0.070	0.031	0.01	0.00	1.00
	Truthfulness × Normativity		0.04	0.04	0.06	0.800	0.044	< 0.01	0.00	1.00
	Truthfulness × Perspective		0.55	0.98	0.32	0.324	0.013	< 0.0	0.00	1.001
	Normativity × Perspective		0.45	0.80	0.37	0.372	0.050	< 0.01	0.00	1.00
	Truthfulness × Normativity × Perspective		0.16	0.16	0.29	0.590	0.025	< 0.01	0.00	1.00
	Residuals	230	129.17	0.56						
**Dishonesty**										
	Truthfulness	1	1.34	1.34	2.10	0.149	0.050	< 0.01	0.00	1.00
	Normativity		0.00	0.00	0.00	0.987	0.044	< 0.01	0.00	1.00
	Perspective		13.52	13.52	21.12	$<$ **0.001**	**0.013**	0.08	0.04	1.00
	Truthfulness × Normativity		1.68	1.68	2.62	0.107	0.038	0.01	0.00	1.00
	Truthfulness × Perspective		0.21	0.21	0.33	0.564	0.019	< 0.01	0.00	1.00
	Normativity × Perspective		0.02	0.02	0.04	0.851	0.031	< 0.01	0.00	1.00

Continued on next page

Table C1

Experiment 1: Full per protocol ANOVA results (continued)

									95% CI	
DV	Effect	df	SS	MS	F	p	BH α	η^2_p	Lower	Upper
	Truthfulness × Normativity × Perspective		0.58	0.58	0.90	0.342	0.025	<0.01	0.00	1.00
	Residuals	230	147.26	0.64						
**Expansion of Power**										
	Truthfulness	1	2.22	2.22	1.70	0.194	0.031	< 0.01	0.00	1.00
	Normativity		0.13	0.13	0.10	0.756	0.050	< 0.01	0.00	1.00
	Perspective		26.27	26.27	20.10	**< 0.001**	**0.013**	0.08	0.03	1.00
	Truthfulness × Normativity		0.00	0.00	0.00	0.956	0.044	< 0.01	0.00	1.00
	Truthfulness × Perspective		1.20	1.20	0.92	0.338	0.019	< 0.01	0.00	1.00
	Normativity × Perspective		0.01	0.01	0.01	0.937	0.038	< 0.01	0.00	1.00
	Truthfulness × Normativity × Perspective		4.62	4.62	3.54	0.061	0.025	0.02	0.00	1.00
	Residuals	230	300.60	1.31						
**Perceived honesty**										
	Truthfulness	1	30.42	30.42	20.49	**<0.001**	**0.019**	0.08	0.03	1.00
	Normativity		53.74	53.74	36.19	**<0.001**	**0.013**	0.14	0.07	1.00
	Perspective		11.60	11.60	7.81	**0.006**	**0.025**	0.03	0.01	1.00
	Truthfulness × Normativity		7.43	7.43	5.01	**0.026**	**0.031**	0.02	0.00	1.00
	Truthfulness × Perspective		0.41	0.41	0.28	0.599	0.044	< 0.01	0.00	1.00
	Normativity × Perspective		8.98	8.98	6.05	**0.014**	**0.025**	0.03	0.00	1.00
	Truthfulness × Normativity × Perspective		3.20	3.20	2.16	0.143	0.038	< 0.01	0.00	1.00

Continued on next page

Table C1

Experiment 1: Full per protocol ANOVA results (continued)

									95% CI	
DV	Effect	df	SS	MS	F	p	BH α	η^2_p	Lower	Upper
	Residuals	230	341.52	1.48						
**Likeability**										
	Truthfulness	1	0.06	0.06	0.04	0.848	0.038	< 0.01	0.00	1.00
	Normativity		32.30	32.30	18.69	< **0.001**	**0.013**	0.08	0.03	1.00
	Perspective		6.52	6.52	3.77	0.053	0.031	0.02	0.00	1.00
	Truthfulness × Normativity		0.24	0.24	0.14	0.711	0.044	< 0.01	0.00	1.00
	Truthfulness × Perspective		1.22	1.22	0.71	0.401	0.050	< 0.01	0.00	1.00
	Normativity × Perspective		0.47	0.47	0.27	0.604	0.019	< 0.01	0.00	1.00
	Truthfulness × Normativity × Perspective		0.90	0.90	0.52	0.472	0.025	< 0.01	0.00	1.00
	Residuals	230	397.49	1.73						

Note. BH stands for Benjamini-Hochberg. This table presents full per protocol ANOVA results from Experiment 1. *Listwise N = 238.*

Table C2

Experiment 2: Full per protocol ANOVA results

									95% CI	
DV	Effect	df	SS	MS	F	p	BH α	η_p^2	Lower	Upper
**Incitement**										
	Truthfulness	1	18.21	18.21	15.73	<**0.001**	**0.013**	0.05	0.02	1.00
	Normativity		3.61	3.61	3.12	0.079	0.044	0.01	0.00	1.00
	Perspective		14.64	14.64	12.65	< **0.001**	**0.019**	0.04	0.01	1.00
	Truthfulness × Normativity		0.99	0.99	0.85	0.356	0.050	< 0.01	0.00	1.00
	Truthfulness × Perspective		1.11	1.11	0.96	0.328	0.031	< 0.01	0.00	1.00
	Normativity × Perspective		2.66	2.66	2.30	0.131	0.025	< 0.01	0.00	1.00
	Truthfulness × Normativity × Perspective		0.15	0.15	0.13	0.719	0.038	< 0.01	0.00	1.00
	Residuals	286	331.04	1.16						
**Dishonesty**										
	Truthfulness	1	5.32	5.32	3.96	0.047	0.013	0.01	0.00	1.00
	Normativity		0.98	0.98	0.73	0.394	0.025	< 0.01	0.00	1.00
	Perspective		18.82	18.82	14.03	< **0.001**	**0.050**	0.05	0.01	1.00
	Truthfulness × Normativity		0.13	0.13	0.10	0.755	0.044	< 0.01	0.00	1.00
	Truthfulness × Perspective		4.69	4.69	3.50	0.062	0.019	0.01	0.00	1.00
	Normativity × Perspective		0.28	0.28	0.21	0.647	0.031	< 0.01	0.00	1.00
	Truthfulness × Normativity × Perspective		1.47	1.47	1.10	0.296	0.038	< 0.01	0.00	1.00
	Residuals	286	383.55	1.34						

Continued on next page

Table C2

Experiment 2: Full per protocol ANOVA results (continued)

									95% CI	
DV	Effect	df	SS	MS	F	p	BH α	η_p^2	Lower	Upper
**Expansion of power**										
	Truthfulness	1	6.41	6.41	4.09	0.044	0.012	0.01	0.00	1.00
	Normativity		0.05	0.05	0.03	0.857	0.31	< 0.01	0.00	1.00
	Perspective		25.11	25.11	16.02	< **0.001**	**0.044**	0.05	0.02	1.00
	Truthfulness × Normativity		0.04	0.04	0.02	0.875	0.050	< 0.01	0.00	1.00
	Truthfulness × Perspective		5.72	5.72	3.65	0.057	0.025	0.01	0.00	1.00
	Normativity × Perspective		7.44	7.44	4.75	**0.030**	**0.019**	0.02	0.00	1.00
	Truthfulness × Normativity × Perspective		0.24	0.24	0.15	0.696	0.038	< 0.01	0.00	1.00
	Residuals	286	448.17	1.57						
**Perceived honesty**										
	Truthfulness	1	46.08	46.08	22.49	< **0.001**	**0.013**	0.07	0.03	1.00
	Normativity		18.95	18.95	9.25	**0.003**	**0.019**	0.03	0.01	1.00
	Perspective		0.03	0.03	0.01	0.908	0.050	< 0.01	0.00	1.00
	Truthfulness × Normativity		1.28	1.28	0.62	0.431	0.044	< 0.01	0.00	1.00
	Truthfulness × Perspective		0.11	0.11	0.05	0.82	0.031	< 0.01	0.00	1.00
	Normativity × Perspective		0.31	0.31	0.15	0.70	0.038	< 0.01	0.00	1.00
	Truthfulness × Normativity × Perspective		1.40	1.40	0.68	0.410	0.025	< 0.01	0.00	1.00
	Residuals	286	585.97	2.05						
**Likeability**										

Continued on next page

Table C2

Experiment 2: Full per protocol ANOVA results (continued)

									95% CI	
DV	Effect	df	SS	MS	F	p	BH α	η^2_p	Lower	Upper
	Truthfulness	1	17.01	17.01	9.73	**0.002**	**0.013**	0.03	0.01	1.00
	Normativity		48.69	48.69	27.84	< **0.001**	**0.006**	0.09	0.04	1.00
	Perspective		0.18	0.18	0.10	0.750	0.038	< 0.01	0.00	1.00
	Truthfulness × Normativity		0.83	0.83	0.47	0.492	0.050	< 0.01	0.00	1.00
	Truthfulness × Perspective		0.01	0.01	0.01	0.943	0.044	< 0.01	0.00	1.00
	Normativity × Perspective		1.41	1.41	0.81	0.369	0.025	< 0.01	0.00	1.00
	Truthfulness × Normativity × Perspective		0.61	0.61	0.35	0.554	0.038	< 0.01	0.00	1.00
	Residuals	286	500.15	1.75						

Note. BH stands for Benjamini-Hochberg. This table presents full per protocol ANOVA results from experiment 2.

Listwise N = 294.

Table C3

Experiment 3: Full per protocol ANOVA results

									95% CI	
DV	Effect	df	SS	MS	F	p	BH α	η_p^2	Lower	Upper
**Incitement**										
	Truthfulness	1	7.77	7.77	6.60	**0.011**	**0.044**	0.01	0.00	1.00
	Normativity		0.06	0.06	0.05	0.818	0.025	< 0.01	0.00	1.00
	Perspective		25.40	25.40	21.58	< **0.001**	**0.013**	0.01	0.02	1.00
	Truthfulness × Normativity		2.73	2.73	2.32	0.128	0.019	< 0.01	0.00	1.00
	Truthfulness × Perspective		0.92	0.92	0.78	0.377	0.050	< 0.01	0.00	1.00
	Normativity × Perspective		0.06	0.06	0.05	0.817	0.031	< 0.01	0.00	1.00
	Truthfulness × Normativity × Perspective		0.28	0.28	0.24	0.625	0.038	< 0.01	0.00	1.00
	Residuals	441	519.11	1.18						
**Dishonesty**										
	Truthfulness	1	0.78	0.78	0.52	0.473	0.025	< 0.01	0.00	1.00
	Normativity		5.33	5.33	3.51	0.062	0.044	< 0.01	0.00	1.00
	Perspective		18.08	18.08	11.90	< **0.001**	**0.013**	0.03	0.01	1.00
	Truthfulness × Normativity		0.01	0.01	0.01	0.927	0.031	< 0.01	0.00	1.00
	Truthfulness × Perspective		3.50	3.50	2.31	0.130	0.019	< 0.01	0.00	1.00
	Normativity × Perspective		0.01	0.01	0.01	0.932	0.050	< 0.01	0.00	1.00
	Truthfulness × Normativity × Perspective		0.27	0.27	0.18	0.671	0.038	< 0.01	0.00	1.00
	Residuals	441	670.11	1.52						

Continued on next page

Table C3

Experiment 3: Full per protocol ANOVA results (continued)

									95% CI	
DV	Effect	df	SS	MS	F	p	BH α	η_p^2	Lower	Upper
**Expansion of power**										
	Truthfulness	1	8.26	8.26	5.34	0.021	0.019	0.01	0.00	1.00
	Normativity		3.39	3.39	2.19	0.139	0.044	< 0.01	0.00	1.00
	Perspective		33.64	33.64	21.75	< **0.001**	**0.013**	0.05	0.02	1.00
	Truthfulness × Normativity		0.23	0.23	0.15	0.702	0.050	< 0.01	0.00	1.00
	Truthfulness × Perspective		2.04	2.04	1.32	0.251	0.025	< 0.01	0.00	1.00
	Normativity × Perspective		1.31	1.31	0.85	0.358	0.038	< 0.01	0.00	1.00
	Truthfulness × Normativity × Perspective		0.27	0.27	0.17	0.677	0.031	< 0.01	0.00	1.00
	Residuals	441	682.05	1.55						
**Perceived honesty**										
	Truthfulness	1	59.63	59.63	28.56	< **0.001**	**0.019**	0.06	0.03	1.00
	Normativity		92.20	92.20	44.16	< **0.001**	**0.013**	0.09	0.05	1.00
	Perspective		9.99	9.99	4.78	0.028	0.025	0.01	0.00	1.00
	Truthfulness × Normativity		0.54	0.54	0.26	0.596	0.031	< 0.01	0.00	1.00
	Truthfulness × Perspective		5.05	5.05	2.42	0.130	0.050	< 0.01	0.00	1.00
	Normativity × Perspective		7.13	7.13	3.42	0.063	0.044	< 0.01	0.00	1.00
	Truthfulness × Normativity × Perspective		1.59	1.59	0.76	0.368	0.038	< 0.01	0.00	1.00
	Residuals	441	920.70	2.09						
**Likeability**										

Continued on next page

Table C3

Experiment 3: Full per protocol ANOVA results (continued)

DV	Effect	df	SS	MS	F	p	BH α	η^2_p	95% CI Lower	95% CI Upper
	Truthfulness	1	3.41	3.41	1.55	0.220	0.050	< 0.01	0.00	1.00
	Normativity		59.03	59.03	26.85	< **0.001**	**0.013**	0.06	0.03	1.00
	Perspective		2.45	2.45	1.11	0.31	0.031	< 0.01	0.00	1.00
	Truthfulness × Normativity		2.09	2.09	0.95	0.328	0.025	< 0.01	0.00	1.00
	Truthfulness × Perspective		1.26	1.26	0.57	0.431	0.044	< 0.01	0.00	1.00
	Normativity × Perspective		10.22	10.22	4.65	0.031	0.019	0.01	0.00	1.00
	Truthfulness × Normativity × Perspective		0.68	0.68	0.31	0.571	0.038	< 0.01	0.00	1.00
	Residuals	441	969.57	2.20						

Note. BH stands for Benjamini-Hochberg. This table presents full per protocol ANOVA results from experiment 3.

Listwise N = 449.

Table C4

Experiment 4: Full per protocol ANOVA results

									95% CI	
DV	Effect	df	SS	MS	F	p	BH α	η^2_p	Lower	Upper
**Incitement**										
	Truthfulness	1	43.30	43.30	35.15	< **0.001**	**0.006**	0.06	0.03	1.00
	Normativity		6.19	6.19	5.02	**0.03**	**0.019**	< 0.01	0.00	1.00
	Perspective		35.41	35.41	28.75	< **0.001**	**0.013**	0.05	0.01	1.00
	Truthfulness × Normativity		1.08	1.08	0.88	0.35	0.038	< 0.01	0.03	1.00
	Truthfulness × Perspective		1.20	1.20	0.98	0.32	0.025	< 0.01	0.03	1.00
	Normativity × Perspective		1.18	1.18	0.96	0.33	0.031	< 0.01	0.03	1.00
	Truthfulness × Normativity × Perspective		0.38	0.38	0.31	0.58	0.043	< 0.01	0.03	1.00
	Residuals	550	677.56	1.23						
**Dishonesty**										
	Truthfulness	1	0.25	0.25	0.15	0.70	0.043	< 0.01	0.00	1.00
	Normativity		0.64	0.64	0.40	0.53	0.038	< 0.01	0.00	1.00
	Perspective		31.88	31.88	19.63	< **0.001**	**0.006**	0.03	0.01	1.00
	Truthfulness × Normativity		1.62	1.62	1.00	0.32	0.019	< 0.01	0.00	1.00
	Truthfulness × Perspective		2.19	2.19	1.35	0.25	0.013	< 0.01	0.00	1.00
	Normativity × Perspective		1.03	1.03	0.63	0.43	0.025	< 0.01	0.00	1.00
	Truthfulness × Normativity × Perspective		0.71	0.71	0.44	0.51	0.031	< 0.01	0.00	1.00
	Residuals	550	893.39	1.62						

Continued on next page

Table C4

Experiment 4: Full per protocol ANOVA results (continued)

									95% CI	
DV	Effect	df	SS	MS	F	p	BH α	η^2_p	Lower	Upper
**Expansion of power**										
	Truthfulness	1	14.19	14.19	10.32	**0.001**	**0.013**	0.02	0.01	1.00
	Normativity		0.16	0.16	0.12	0.73	0.038	< 0.01	0.00	1.00
	Perspective		39.16	39.16	28.47	< **0.001**	**0.006**	0.05	0.01	1.00
	Truthfulness × Normativity		10.44	10.44	7.59	**0.006**	**0.019**	0.01	0.00	1.00
	Truthfulness × Perspective		1.03	1.03	0.75	0.39	0.031	< 0.01	0.00	1.00
	Normativity × Perspective		0.10	0.10	0.07	0.79	0.044	< 0.01	0.00	1.00
	Truthfulness × Normativity × Perspective		1.12	1.12	0.81	0.37	0.025	< 0.01	0.00	1.00
	Residuals	550	756.53	1.38						
**Perceived honesty**										
	Truthfulness	1	111.36	111.36	58.48	< **0.001**	**0.006**	0.10	0.07	1.00
	Normativity		43.22	43.22	22.70	< **0.001**	**0.013**	0.04	0.01	1.00
	Perspective		21.40	21.40	11.24	< **0.001**	**0.019**	0.02	0.01	1.00
	Truthfulness × Normativity		0.33	0.33	0.17	0.68	0.044	< 0.01	0.00	1.00
	Truthfulness × Perspective		1.22	1.22	0.64	0.42	0.038	< 0.01	0.00	1.00
	Normativity × Perspective		11.57	11.57	6.07	**0.014**	**0.031**	0.01	0.00	1.00
	Truthfulness × Normativity × Perspective		16.51	16.51	8.67	**0.003**	**0.025**	0.02	0.00	1.00
	Residuals	550	1047.27	1.90						
**Likeability**										

Continued on next page

Table C4

Experiment 4: Full per protocol ANOVA results (continued)

									95% CI	
DV	Effect	df	SS	MS	F	p	BH α	η^2_p	Lower	Upper
	Truthfulness	1	17.73	17.73	9.36	$<$ **0.001**	**0.013**	0.02	0.00	1.00
	Normativity		42.87	42.87	22.62	$<$ **0.001**	**0.006**	0.05	0.01	1.00
	Perspective		8.16	8.16	4.31	0.04	0.019	$<$ 0.01	0.00	1.00
	Truthfulness × Normativity		0.53	0.53	0.28	0.60	0.038	$<$ 0.01	0.00	1.00
	Truthfulness × Perspective		7.73	7.73	4.08	**0.04**	**0.003**	$<$ 0.01	0.00	1.00
	Normativity × Perspective		0.03	0.03	0.02	0.90	0.044	$<$ 0.01	0.00	1.00
	Truthfulness × Normativity × Perspective		4.88	4.88	2.57	0.11	0.031	$<$ 0.01	0.00	1.00
	Residuals	550	1042.47	1.90						

Note. BH stands for Benjamini-Hochberg. This table presents full per protocol ANOVA results from Experiment 4.

Listwise N = 558.

Table C5

Experiment 4: Full per protocol ANOVA results for right-wing participants

									95% CI	
DV	Effect	df	SS	MS	F	p	BH α	η^2_{p}	Lower	Upper
**Incitement**										
	Truthfulness	1	20.56	20.56	9.80	< **0.001**	**0.013**	0.07	0.03	1.00
	Normativity		1.39	1.39	1.34	0.248	0.025	< 0.01	0.00	1.00
	Perspective		5.61	5.61	5.40	0.021	0.019	0.02	0.00	1.00
	Truthfulness × Normativity		0.46	0.46	0.45	0.506	0.031	< 0.01	0.00	1.00
	Truthfulness × Perspective		0.06	0.06	0.06	0.803	0.038	< 0.01	0.00	1.00
	Normativity × Perspective		< 0.01	< 0.01	< 0.01	0.951	0.050	< 0.01	0.00	1.00
	Truthfulness × Normativity × Perspective		0.16	0.16	0.16	0.691	0.044	< 0.01	0.00	1.00
	Residuals	263	272.998	1.038						
**Dishonesty**										
	Truthfulness	1	< 0.01	< 0.01	< 0.01	0.954	0.038	< 0.01	0.00	1.00
	Normativity		0.03	0.03	0.02	0.897	0.044	< 0.01	0.00	1.00
	Perspective		2.32	2.32	1.40	0.237	0.050	< 0.01	0.00	1.00
	Truthfulness × Normativity		2.61	2.61	1.58	0.211	0.031	< 0.01	0.00	1.00
	Truthfulness × Perspective		< 0.01	< 0.01	< 0.01	0.988	0.025	< 0.01	0.00	1.00
	Normativity × Perspective		1.25	1.25	0.76	0.386	0.013	< 0.01	0.00	1.00
	Truthfulness × Normativity × Perspective		0.73	0.73	0.44	0.508	0.019	< 0.01	0.00	1.00
	Residuals	263	435.1	1.655						

Continued on next page

Table C5

Experiment 4: Full per protocol ANOVA results for right-wing participants (continued)

									95% CI	
DV	Effect	df	SS	MS	F	p	BH α	η^2_{p}	Lower	Upper
**Expansion of power**										
	Truthfulness	1	3.20	3.20	2.41	0.122	0.044	< 0.01	0.00	1.00
	Normativity		< 0.01	< 0.01	< 0.01	0.942	0.019	< 0.01	0.00	1.00
	Perspective		14.34	4.34	10.80	**0.005**	**0.025**	0.04	0.01	1.00
	Truthfulness × Normativity		10.67	10.67	8.04	**0.005**	**0.013**	0.03	0.01	1.00
	Truthfulness × Perspective		3.09	3.09	2.33	0.128	0.031	< 0.01	0.00	1.00
	Normativity × Perspective		< 0.01	< 0.01	< 0.01	0.988	0.050	< 0.01	0.00	1.00
	Truthfulness × Normativity × Perspective		0.30	0.30	0.23	0.636	0.038	< 0.01	0.00	1.00
	Residuals	263	349.215	1.328						
**Perceived honesty**										
	Truthfulness	1	61.49	61.49	40.97	< **0.001**	**0.013**	0.13	0.08	1.00
	Normativity		26.26	26.26	17.50	< **0.001**	**0.050**	0.06	0.02	1.00
	Perspective		10.78	10.78	7.18	**0.008**	**0.025**	0.03	0.00	1.00
	Truthfulness × Normativity		0.98	0.98	0.66	0.419	0.038	< 0.01	0.00	1.00
	Truthfulness × Perspective		1.82	1.82	1.21	0.271	0.044	< 0.01	0.00	1.00
	Normativity × Perspective		9.31	9.31	6.20	**0.013**	**0.019**	0.02	0.00	1.00
	Truthfulness × Normativity × Perspective		4.47	4.47	2.98	0.086	0.031	0.01	0.00	1.00
	Residuals	263	394.680	1.501						
**Likeability**										

Continued on next page

Table C5

Experiment 4: Full per protocol ANOVA results for right-wing participants (continued)

									95% CI	
DV	Effect	df	SS	MS	F	p	BH α	η^2_{p}	Lower	Upper
	Truthfulness		22.53	22.53	10.76	**0.001**	**0.025**	0.04	0.01	1.00
	Normativity		22.21	22.21	10.60	**0.001**	**0.013**	0.04	0.01	1.00
	Perspective		2.616	2.616	1.249	0.265	0.044	< 0.01	0.00	1.00
	Truthfulness × Normativity		3.343	3.343	1.596	0.208	0.050	< 0.01	0.00	1.00
	Truthfulness × Perspective		12.690	12.690	6.059	**0.014**	**0.019**	0.02	0.00	1.00
	Normativity × Perspective		0.005	0.005	0.002	0.961	0.031	< 0.01	0.00	1.00
	Truthfulness × Normativity × Perspective		0.896	0.896	0.428	0.514	0.038	0.02	0.00	1.00
	Residuals	263	550.800	2.094						

Note. BH stands for Benjamini-Hochberg. This table presents full per protocol ANOVA results for right-wing participants from Experiment 4. *Listwise N = 549.*

Appendix D

Full per protocol ANCOVA tables for all experiments

Table D1

Experiment 2: Full per protocol ANCOVA results

DV	Effect	df	SS	MS	F	p	BH α	η^2_p	95% CI Lower	95% CI Upper
**Incitement**										
	Truthfulness	1	15.25	15.25	12.86	$<$ **0.001**	**0.006**	< 0.01	0.01	1.00
	Normativity		2.43	2.43	2.05	0.153	0.019	< 0.01	0.00	1.00
	Perspective		12.09	12.09	10.20	**0.002**	**0.013**	< 0.01	0.01	1.00
	E2IS		0.30	0.30	0.26	0.614	0.044	< 0.01	0.00	1.00
	Truthfulness × Normativity		1.65	1.65	1.39	0.239	0.031	< 0.01	0.00	1.00
	Truthfulness × Perspective		1.49	1.49	1.25	0.264	0.038	< 0.01	0.00	1.00
	Normativity × Perspective		2.36	2.36	1.99	0.160	0.025	< 0.01	0.00	1.00
	Truthfulness × Normativity × Perspective		0.00	0.00	0.00	0.952	0.050	< 0.01	0.00	1.00
	Residuals	259	306.99	1.19						
**Dishonesty**										
	Truthfulness	1	3.40	3.40	2.63	0.106	0.025	< 0.01	0.00	1.00
	Normativity		0.50	0.50	0.39	0.535	0.044	< 0.01	0.00	1.00
	Perspective		16.57	16.57	12.82	$<$ **0.001**	**0.006**	< 0.01	0.01	1.00
	E2IS		3.68	3.68	2.85	0.093	0.019	< 0.01	0.00	1.00
	Truthfulness × Normativity		0.93	0.93	0.72	0.396	0.038	< 0.01	0.00	1.00

Continued on next page

Table D1

Experiment 2: Full per protocol ANCOVA results (continued)

									95% CI	
DV	Effect	df	SS	MS	F	p	BH α	η^2_{p}	Lower	Upper
	Truthfulness × Perspective		5.60	5.60	4.33	**0.038**	**0.013**	< 0.01	0.00	1.00
	Normativity × Perspective		0.22	0.22	0.17	0.677	0.050	< 0.01	0.00	1.00
	Truthfulness × Normativity × Perspective		2.02	2.02	1.56	0.212	0.031	< 0.01	0.00	1.00
	Residuals	259	334.62	1.29						
**Expansion of Power**										
	Truthfulness	1	4.00	4.00	2.62	0.107	0.031	< 0.01	0.00	1.00
	Normativity		0.19	0.19	0.12	0.727	0.038	< 0.01	0.00	1.00
	Perspective		24.54	24.54	16.11	< **0.001**	**0.006**	< 0.01	0.02	1.00
	E2IS		11.66	11.66	7.66	**0.006**	**0.013**	< 0.01	0.00	1.00
	Truthfulness × Normativity		0.00	0.00	0.00	0.979	0.044	< 0.01	0.00	1.00
	Truthfulness × Perspective		6.86	6.86	4.50	0.035	0.025	< 0.01	0.00	1.00
	Normativity × Perspective		7.49	7.49	4.91	0.028	0.019	< 0.01	0.00	1.00
	Truthfulness × Normativity × Perspective		0.00	0.00	0.00	0.993	0.050	< 0.01	0.00	1.00
	Residuals	259	394.59	1.52						
**Perceived Honesty**										
	Truthfulness	1	42.34	42.34	20.86	< **0.001**	**0.006**	< 0.01	0.03	1.00
	Normativity		14.05	14.05	6.92	**0.009**	**0.013**	< 0.01	0.00	1.00
	Perspective		0.53	0.53	0.26	0.609	0.031	< 0.01	0.00	1.00
	E2IS		5.59	5.59	2.75	0.098	0.019	< 0.01	0.00	1.00

Continued on next page

Table D1

Experiment 2: Full per protocol ANCOVA results (continued)

									95% CI	
DV	Effect	df	SS	MS	F	p	BH α	η^2_p	Lower	Upper
	Truthfulness × Normativity		1.46	1.46	0.72	0.397	0.025	< 0.01	0.00	1.00
	Truthfulness × Perspective		0.01	0.01	0.01	0.932	0.044	< 0.01	0.00	1.00
	Normativity × Perspective		0.02	0.02	0.01	0.928	0.050	< 0.01	0.00	1.00
	Truthfulness × Normativity × Perspective		0.29	0.29	0.14	0.704	0.038	< 0.01	0.00	1.00
	Residuals	259	525.75	2.03						
**Likeability**										
	Truthfulness	1	16.67	16.67	9.54	**0.002**	**0.013**	< 0.01	0.00	1.00
	Normativity		38.83	38.83	22.22	< **0.001**	**0.006**	< 0.01	0.03	1.00
	Perspective		0.01	0.01	0.00	0.945	0.050	< 0.01	0.00	1.00
	E2IS		3.35	3.35	1.92	0.167	0.019	< 0.01	0.00	1.00
	Truthfulness × Normativity		0.50	0.50	0.29	0.594	0.031	< 0.01	0.00	1.00
	Truthfulness × Perspective		0.01	0.01	0.01	0.937	0.044	< 0.01	0.00	1.00
	Normativity × Perspective		1.26	1.26	0.72	0.396	0.025	< 0.01	0.00	1.00
	Truthfulness × Normativity × Perspective		0.39	0.39	0.22	0.637	0.038	< 0.01	0.00	1.00
	Residuals	259	452.50	1.75						

Note. BH stands for Benjamini-Hochberg. This table presents full per protocol ANCOVA results from Experiment 2.

Listwise N = 294.

Table D2

Experiment 3: Full per protocol ANCOVA results for E2IS

									95% CI	
DV	Effect	df	SS	MS	F	p	BH α	η_p^2	Lower	Upper
**Incitement**										
	Truthfulness	1	7.77	7.77	6.62	**0.010**	**0.013**	< 0.01	0.00	1.00
	Normativity		0.06	0.06	0.05	0.818	0.044	< 0.01	0.00	1.00
	Perspective		25.40	25.40	21.65	< 0.001	0.006	< 0.01	0.02	1.00
	E2IS		3.13	3.13	2.67	0.103	0.019	< 0.01	0.00	1.00
	Truthfulness × Normativity		2.55	2.55	2.17	0.141	0.025	< 0.01	0.00	1.00
	Truthfulness × Perspective		0.85	0.85	0.72	0.395	0.031	< 0.01	0.00	1.00
	Normativity × Perspective		0.04	0.04	0.04	0.849	0.050	< 0.01	0.00	1.00
	Truthfulness × Normativity × Perspective		0.33	0.33	0.28	0.594	0.038	< 0.01	0.00	1.00
	Residuals	440	516.21	1.17						
**Dishonesty**										
	Truthfulness	1	0.78	0.78	0.52	0.473	0.031	< 0.01	0.00	1.00
	Normativity		5.33	5.33	3.51	0.062	0.013	< 0.01	0.00	1.00
	Perspective		18.08	18.08	11.90	< **0.001**	**0.006**	< 0.01	0.01	1.00
	E2IS		1.85	1.85	1.22	0.270	0.025	< 0.01	0.00	1.00
	Truthfulness × Normativity		0.00	0.00	0.00	0.956	0.050	< 0.01	0.00	1.00
	Truthfulness × Perspective		3.39	3.39	2.23	0.136	0.019	< 0.01	0.00	1.00
	Normativity × Perspective		0.02	0.02	0.01	0.909	0.044	< 0.01	0.00	1.00

Continued on next page

Table D2

Experiment 3: Full per protocol ANCOVA results for E2IS (continued)

									95% CI	
DV	Effect	df	SS	MS	F	p	BH α	η^2_p	Lower	Upper
	Truthfulness × Normativity × Perspective		0.24	0.24	0.16	0.693	0.038	< 0.01	0.00	1.00
	Residuals	440	668.41	1.52						
**Expansion of Power**										
	Truthfulness	1	8.26	8.26	5.39	0.021	0.013	< 0.01	0.00	1.00
	Normativity		3.39	3.39	2.22	0.137	0.025	< 0.01	0.00	1.00
	Perspective		33.64	33.64	21.96	< **0.001**	**0.006**	< 0.01	0.02	1.00
	E2IS		8.07	8.07	5.27	0.022	0.019	< 0.01	0.00	1.00
	Truthfulness × Normativity		0.15	0.15	0.09	0.758	0.050	< 0.01	0.00	1.00
	Truthfulness × Perspective		1.86	1.86	1.21	0.271	0.031	< 0.01	0.00	1.00
	Normativity × Perspective		1.48	1.48	0.97	0.326	0.038	< 0.01	0.00	1.00
	Truthfulness × Normativity × Perspective		0.36	0.36	0.23	0.629	0.044	< 0.01	0.00	1.00
	Residuals	440	673.97	1.53						
**Perceived Honesty**										
	Truthfulness	1	56.04	56.04	27.88	< **0.001**	**0.013**	< 0.01	0.03	1.00
	Normativity		90.43	90.43	44.99	< **0.001**	**0.006**	< 0.01	0.05	1.00
	Perspective		9.82	9.82	4.89	0.028	**0.019**	< 0.01	0.00	1.00
	E2IS		8.93	8.93	4.44	0.036	**0.025**	< 0.01	0.00	1.00
	Truthfulness × Normativity		0.43	0.43	0.21	0.644	0.050	< 0.01	0.00	1.00
	Truthfulness × Perspective		4.97	4.97	2.47	0.116	0.038	< 0.01	0.00	1.00

Continued on next page

Table D2

Experiment 3: Full per protocol ANCOVA results for E2IS (continued)

									95% CI	
DV	Effect	df	SS	MS	F	p	BH α	η^2_p	Lower	Upper
	Normativity × Perspective		6.62	6.62	3.29	0.070	0.031	< 0.01	0.00	1.00
	Truthfulness × Normativity × Perspective		1.44	1.44	0.72	0.398	0.044	< 0.01	0.00	1.00
	Residuals	440	884.29	2.01						
**Likeability**										
	Truthfulness	1	3.29	3.29	1.51	0.220	0.025	< 0.01	0.00	1.00
	Normativity		58.35	58.35	26.78	< **0.001**	**0.006**	< 0.01	0.03	1.00
	Perspective		2.22	2.22	1.02	0.313	0.031	< 0.01	0.00	1.00
	E2IS		4.66	4.66	2.14	0.144	0.019	< 0.01	0.00	1.00
	Truthfulness × Normativity		1.89	1.89	0.87	0.352	0.038	< 0.01	0.00	1.00
	Truthfulness × Perspective		1.47	1.47	0.68	0.411	0.044	< 0.01	0.00	1.00
	Normativity × Perspective		9.87	9.87	4.53	0.034	0.013	< 0.01	0.00	1.00
	Truthfulness × Normativity × Perspective		0.61	0.61	0.28	0.597	0.050	< 0.01	0.00	1.00
	Residuals	440	958.53	2.18						

Note. BH stands for Benjamini-Hochberg. This table presents full per protocol ANCOVA results for E2IS from Experiment 3. *Listwise N = 449.*

Table D3

Experiment 4: Full per protocol ANCOVAs for E2IS

DV	Effect	df	SS	MS	F	p	BH α	η^2_p	95% CI	
									Lower	Upper
**Incitement**										
	Truthfulness	1	43.30	43.30	35.76	<**0.001**	**0.006**	0.06	0.03	1.00
	Normativity		6.19	6.19	5.11	0.024	0.022	< 0.01	0.00	1.00
	Perspective		35.41	35.41	29.24	<**0.001**	**0.011**	0.05	0.02	1.00
	E2IS		12.71	12.71	10.50	<**0.001**	**0.017**	0.02	0.00	1.00
	Truthfulness × Normativity		1.23	1.23	1.02	0.314	0.033	< 0.01	0.00	1.00
	Truthfulness × Perspective		1.04	1.04	0.86	0.354	0.039	< 0.01	0.00	1.00
	Normativity × Perspective		1.30	1.30	1.072	0.301	0.028	< 0.01	0.00	1.00
	Truthfulness × Normativity × Perspective		0.21	0.21	0.17	0.676	0.044	0.00	0.00	1.00
	Residuals	549	664.91	1.21						
**Dishonesty**										
	Truthfulness	1	0.25	0.25	0.16	0.694	0.044	< 0.01	0.00	1.00
	Normativity		0.64	0.64	0.40	0.525	0.033	< 0.01	0.00	1.00
	Perspective		31.88	31.88	20.09	<**0.001**	**0.006**	0.04	0.01	1.00
	E2IS		21.69	21.69	13.67	<**0.001**	**0.011**	0.02	0.01	1.00
	Truthfulness × Normativity		1.86	1.86	1.17	0.279	0.022	< 0.01	0.00	1.00
	Truthfulness × Perspective		2.49	2.49	1.57	0.211	0.017	< 0.01	0.00	1.00
	Normativity × Perspective		1.18	1.18	0.74	0.389	0.028	< 0.01	0.00	1.00

Continued on next page

Table D3

Experiment 4: Full per protocol ANCOVA for E2IS (continued)

									95% CI	
DV	Effect	df	SS	MS	F	p	BH α	η^2_p	Lower	Upper
	Truthfulness × Normativity × Perspective		0.40	0.40	0.25	0.617	0.039	0.00	0.00	1.00
	Residuals	549	871.32	1.59						
**Expansion of Power**										
	Truthfulness	1	14.20	14.20	10.51	**0.001**	**0.017**	0.02	0.00	1.00
	Normativity		0.16	0.16	0.12	0.728	0.039	< 0.01	0.00	1.00
	Perspective		39.16	39.16	29.00	**0.001**	**0.006**	0.05	0.02	1.00
	E2IS		14.35	14.35	10.62	**0.001**	**0.011**	0.02	0.00	1.00
	Truthfulness × Normativity		10.92	10.92	8.09	**0.005**	**0.022**	0.01	0.00	1.00
	Truthfulness × Perspective		0.88	0.88	0.65	0.420	0.033	< 0.01	0.00	1.00
	Normativity × Perspective		0.14	0.14	0.10	0.747	0.044	< 0.01	0.00	1.00
	Truthfulness × Normativity × Perspective		1.51	1.51	1.12	0.290	0.028	0.002	0.00	1.00
	Residuals	549	741.418	1.350						
**Perceived honesty**										
	Truthfulness	1	111.36	111.36	59.76	$<$ **0.001**	**0.006**	0.10	0.06	1.00
	Normativity		43.22	43.22	23.19	$<$ **0.001**	**0.011**	0.04	0.02	1.00
	Perspective		21.41	21.41	11.49	**0.001**	**0.022**	0.02	0.01	1.00
	E2IS		26.43	26.43	14.18	$<$ **0.001**	**0.017**	0.03	0.01	1.00
	Truthfulness × Normativity		0.23	0.23	0.12	0.727	0.044	< 0.01	0.00	1.00
	Truthfulness × Perspective		1.47	1.47	0.79	0.375	0.039	< 0.01	0.00	1.00

Continued on next page

Table D3

Experiment 4: Full per protocol ANCOVA for E2IS (continued)

DV	Effect	df	SS	MS	F	p	BH α	η_p^2	95% CI Lower	95% CI Upper
	Normativity × Perspective		11.05	11.05	5.93	0.015	0.033	0.01	0.00	1.00
	Truthfulness × Normativity × Perspective		14.74	14.74	7.91	0.005	0.028	0.014	0.00	1.00
	Residuals	549	1022.981	1.863						
**Likeability**										
	Truthfulness	1	17.73	17.73	9.51	**0.002**	**0.017**	0.10	0.06	1.00
	Normativity		42.87	42.87	22.99	< **0.001**	**0.006**	0.04	0.02	1.00
	Perspective		8.16	8.16	4.38	0.037	0.028	0.02	0.01	1.00
	E2IS		18.85	18.85	10.11	**0.002**	**0.011**	0.03	0.01	1.00
	Truthfulness × Normativity		0.66	0.66	0.36	0.55	0.039	< 0.01	0.00	1.00
	Truthfulness × Perspective		8.25	8.25	4.42	0.036	0.022	< 0.01	0.00	1.00
	Normativity × Perspective		0.01	0.01	0.01	0.940	0.044	0.01	0.00	1.00
	Truthfulness × Normativity × Perspective		4.06	4.06	2.18	0.141	0.033	0.004	0.00	1.00
	Residuals	549	1023.814	1.865						

Note. BH stands for Benjamini-Hochberg. This table presents full per protocol ANCOVA results from Experiment 4.

Listwise N = 549.

Table D4

Experiment 4: Full per protocol ANCOVAs for political orientation

DV	Effect	df	SS	MS	F	p	BH α	η^2_p	95% CI Lower	95% CI Upper
**Incitement**										
	Truthfulness	1	43.304	43.304	35.755	< **0.001**	**0.006**	0.06	0.04	1.00
	Normativity		6.19	6.19	5.11	0.024	0.022	< 0.01	0.00	1.00
	Perspective		35.41	35.41	29.24	< **0.001**	**0.011**	0.05	0.03	1.00
	Political orientation		48.54	48.54	42.29	< **0.001**	**0.006**	< 0.01	0.00	1.00
	Truthfulness × Normativity		0.98	0.98	0.86	0.355	0.033	< 0.01	0.00	1.00
	Truthfulness × Perspective		0.58	0.58	0.50	0.478	0.039	< 0.01	0.00	1.00
	Normativity × Perspective		1.15	1.15	1.00	0.317	0.028	< 0.01	0.00	1.00
	Truthfulness × Normativity × Perspective		0.076	0.076	0.066	0.797	0.044	0.00	0.00	1.00
	Residuals	549	630.075	1.148						
**Dishonesty**										
	Truthfulness	1	0.25	0.25	0.15	0.696	0.044	< 0.01	0.00	1.00
	Normativity		0.642	0.642	0.398	0.528	0.033	< 0.01	0.00	1.00
	Perspective		31.88	31.88	19.77	< **0.001**	**0.006**	0.03	0.00	1.00
	Political orientation		8.09	8.09	5.02	0.025	0.011	0.03	0.01	1.00
	Truthfulness × Normativity		1.57	1.57	0.98	0.324	0.022	< 0.01	0.00	1.00
	Truthfulness × Perspective		2.62	2.62	1.63	0.203	0.017	< 0.01	0.00	1.00
	Normativity × Perspective		1.02	1.02	0.63	0.427	0.028	< 0.01	0.00	1.00

Continued on next page

Table D4

Experiment 4: Full per protocol ANCOVA for political orientation (continued)

									95% CI	
DV	Effect	df	SS	MS	F	p	BH α	η^2_p	Lower	Upper
	Truthfulness × Normativity × Perspective		0.49	0.49	0.30	0.583	0.039	0.001	0.00	1.00
	Residuals	549	885.144	1.612						
**Expansion of Power**										
	Truthfulness	1	14.2	14.2	10.51	**0.001**	**0.017**	0.02	0.00	1.00
	Normativity		0.16	0.16	0.12	0.728	0.039	< 0.01	0.00	1.00
	Perspective		39.16	39.16	28.99	< **0.001**	**0.006**	0.05	0.02	1.00
	Political orientation		15.05	15.05	11.15	< **0.001**	**0.011**	0.02	0.01	1.00
	Truthfulness × Normativity		10.26	10.26	7.60	**0.006**	**0.022**	0.01	0.00	1.00
	Truthfulness × Perspective		0.69	0.69	0.51	0.475	0.033	< 0.01	0.00	1.00
	Normativity × Perspective		0.097	0.097	0.072	0.789	0.044	< 0.01	0.00	1.00
	Truthfulness × Normativity × Perspective		1.56	1.56	1.16	0.28	0.028	0.002	0.00	1.00
	Residuals	549	741.553	1.351						
**Perceived Honesty**										
	Truthfulness	1	111.36	111.36	63.56	< **0.001**	**0.006**	0.10	0.07	1.00
	Normativity		43.22	43.22	24.67	< **0.001**	**0.017**	0.04	0.02	1.00
	Perspective		21.41	21.41	12.22	**0.001**	**0.022**	0.02	0.01	1.00
	Political orientation		87.62	87.62	50.01	< **0.001**	**0.011**	0.08	0.05	1.00
	Truthfulness × Normativity		0.41	0.41	0.24	0.628	0.044	< 0.01	0.00	1.00
	Truthfulness × Perspective		2.43	2.43	1.39	0.239	0.039	< 0.01	0.00	1.00

Continued on next page

Table D4

Experiment 4: Full per protocol ANCOVA for political orientation (continued)

DV	Effect	df	SS	MS	F	p	BH α	η^2_p	95% CI	
									Lower	Upper
	Normativity × Perspective		11.68	11.68	6.67	0.010	0.033	0.01	0.00	1.00
	Truthfulness × Normativity × Perspective		12.95	12.95	7.39	**0.007**	**0.028**	0.01	0.00	1.00
	Residuals	549	961.804	1.752						
**Likeability**										
	Truthfulness	1	17.73	17.73	10.46	**0.001**	**0.017**	0.02	0.00	1.00
	Normativity		42.871	42.871	25.295	$<$ **0.001**	**0.011**	0.04	0.02	1.00
	Perspective		8.163	8.163	4.816	0.029	0.028	<0.01	0.00	1.00
	Political orientation		111.04	111.04	65.51	$<$ **0.001**	**0.006**	0.11	0.07	1.00
	Truthfulness × Normativity		0.43	0.43	0.25	0.615	0.039	<0.01	0.00	1.00
	Truthfulness × Perspective		10.85	10.85	6.40	**0.012**	**0.022**	0.01	0.00	1.00
	Normativity × Perspective		0.036	0.036	0.021	0.885	0.044	<0.01	0.00	1.00
	Truthfulness × Normativity × Perspective		2.82	2.82	1.67	0.197	0.033	<0.01	0.00	1.00
	Residuals	549	930.470	1.695						

Note. BH stands for Benjamini-Hochberg. This table presents full per protocol ANCOVA results from Experiment 4.

Listwise $N = 549$.
